# Testicular rupture successfully treated with a tunica vaginalis flap

**DOI:** 10.1002/iju5.12246

**Published:** 2020-12-17

**Authors:** Shuhei Yokokawa, Tadashi Tabei, Kazuki Kobayashi

**Affiliations:** ^1^ Department of Urology Yokosuka Kyosai Hospital Yokosuka Kanagawa Japan

**Keywords:** rupture, scrotum, surgical flaps, surgical procedures, testis

## Abstract

**Introduction:**

Testicular injury with a tunica albuginea tear is typically reconstructed by primary closure. We herein describe the successful use of a tunica vaginalis flap for reconstruction of a ruptured testis for which primary closure was not possible.

**Case presentation:**

A 21‐year‐old man visited our hospital with scrotal swelling after a baseball struck his left testis. Magnetic resonance imaging and ultrasonography indicated a left tunica albuginea tear, and emergency surgery was performed. Primary closure of the tunica albuginea was impossible since a tight closure could cause secondary damage. A vascular pedicle flap was prepared by shaping the tunica vaginalis to replace the tunica albuginea. He was discharged 2 days postoperatively. Ultrasonography showed normal size and blood flow in the ruptured testis at the 2‐week and 3‐month follow‐up.

**Conclusion:**

A testicular vaginalis flap should be considered when primary closure is difficult in cases of testicular rupture with tunica albuginea damage.

**Abbreviations & Acronyms**
FSH: follicle‐stimulating hormone
LH: luteinizing hormone
MRI: magnetic resonance imaging

**Keynote message**

Testicular rupture is a serious injury. The most important goal of surgical treatment for testicular injury is to rescue the affected testis because the functional prognosis is reportedly better than that of orchiectomy. If primary closure is difficult, substitution with the tunica vaginalis could be considered.

## Introduction

Testicular trauma accounts for <1% of all injuries.^1^ While penetrating injuries due to puncture and gunshot wounds are common overseas, most cases in Japan are due to blunt injury sustained during traffic or sports accidents. There are three types of blunt testicular trauma, namely, testicular contusion that maintains the continuity of the tunica albuginea, a ruptured testis with a tear of the tunica albuginea, and testicular prolapse via laceration of the tunica albuginea. Ultrasonography and MRI are useful for the diagnosis of testicular trauma. The diagnostic specificity of ultrasonography is 65–93.5%.^2^, ^3^ In MRI, T2‐weighted imaging is excellent for the evaluation of the tunica albuginea and testicular contents.^4^ Regarding treatment, surgery is generally preferred for cases of tunica albuginea injury. However, the appropriate surgical indication may be difficult to determine. The European Association of Urology and the American Urological Association guidelines recommend surgical exploration for unconfirmed injuries,^5^, ^6^ with procedures including scrotal drainage, debridement, repair of the tunica albuginea, and orchiopexy, or orchiectomy.^7^

Over 90% of ruptured testes can be preserved by appropriate surgeries within 72 hours after onset, whereas delayed diagnosis is likely to result in loss of reproductive and endocrine function.^8^ It is desirable to avoid orchiectomy because seminal or endocrine abnormalities can occur subsequent to orchiectomy.^9^ However, in some cases, primary closure of the tunica albuginea can be difficult. Here, we describe the case of a man with a ruptured testis for which primary closure was not possible, and a tunica vaginalis flap was successfully used.

## Case presentation

### Clinical evaluation

**Fig. 1:**
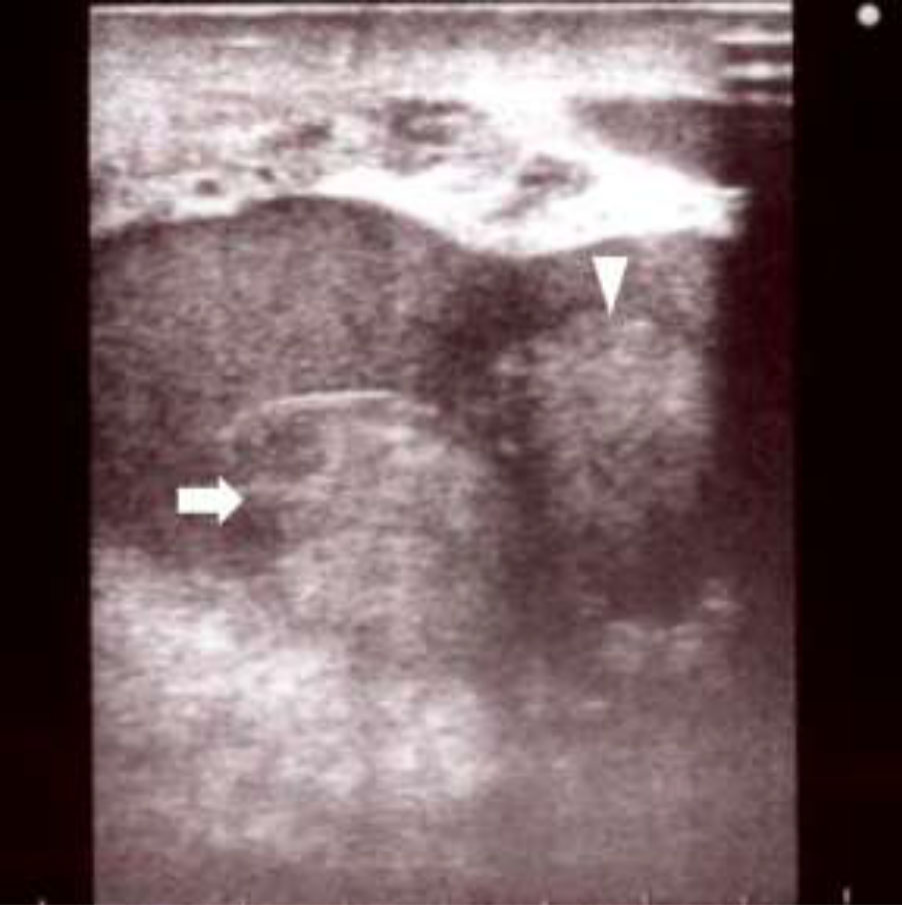
Testicular contents with indistinct perimeter (arrowhead) and heterogeneous internal structure (arrow).

**Fig. 2:**
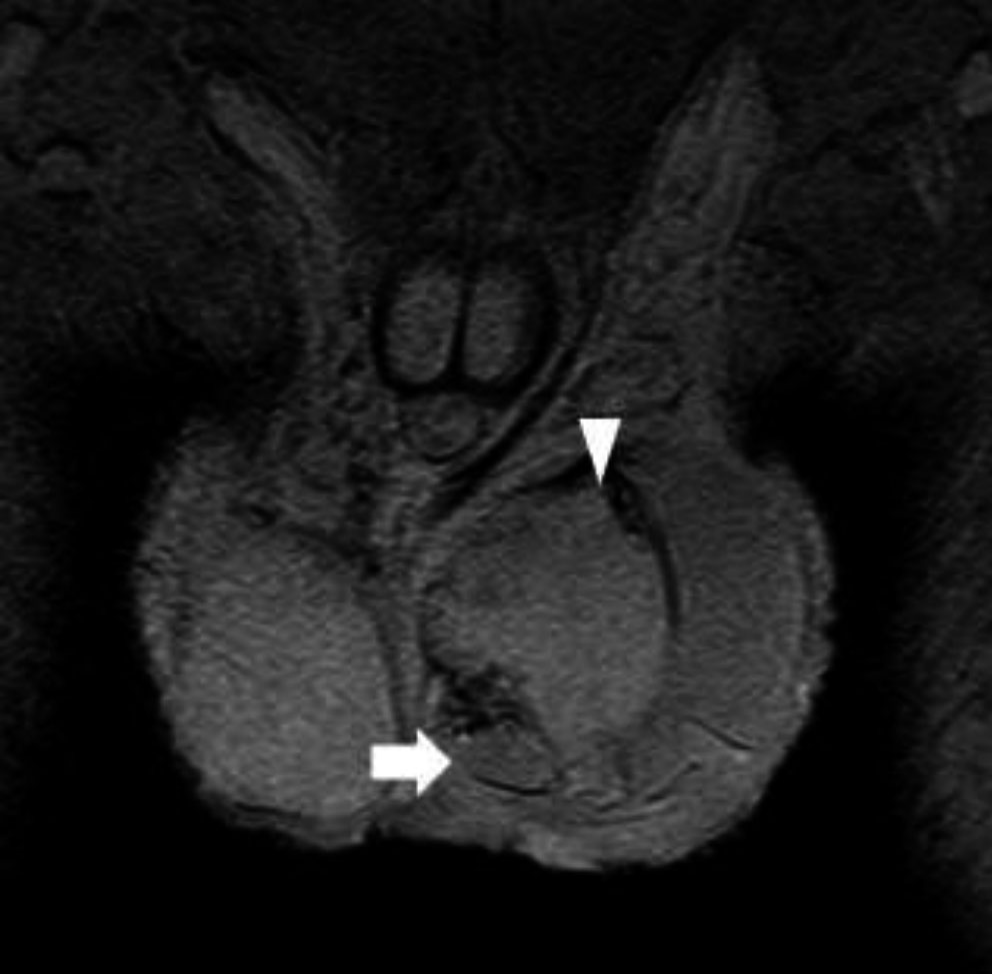
Prolapse of ruptured tunica albuginea (arrow) and testicular contents (arrowhead).

A 21‐year‐old man visited a hospital with scrotal swelling after a baseball struck his left testis. Plain computed tomography revealed no abnormalities. He was treated conservatively with analgesia until he visited the hospital again 5 days later, reporting no improvement. MRI indicated a tear of the left tunica albuginea, and he was referred to our hospital. His scrotum was swollen, and he was in pain at the time of admission. Ultrasonography showed echo‐free space in the left scrotum, including a swollen testis with heterogeneous echogenicity. Doppler ultrasonography revealed no blood flow in the left testis. Blood flow was maintained in the right scrotum, and both epididymides were normal (Fig. 1). MRI indicated swelling of the left testis with surrounding high‐intensity areas and partial disruption of the tunica albuginea (Fig. 2). The preoperative levels of LH, FSH, and testosterone were 6.7 mIU/mL, 13.8 mIU/mL, and 1065.9 ng/dL, respectively. Thus, we suspected testicular rupture with damage to the tunica albuginea. Emergency surgery was performed on the day of admission.

### Surgical findings

**Fig. 3:**
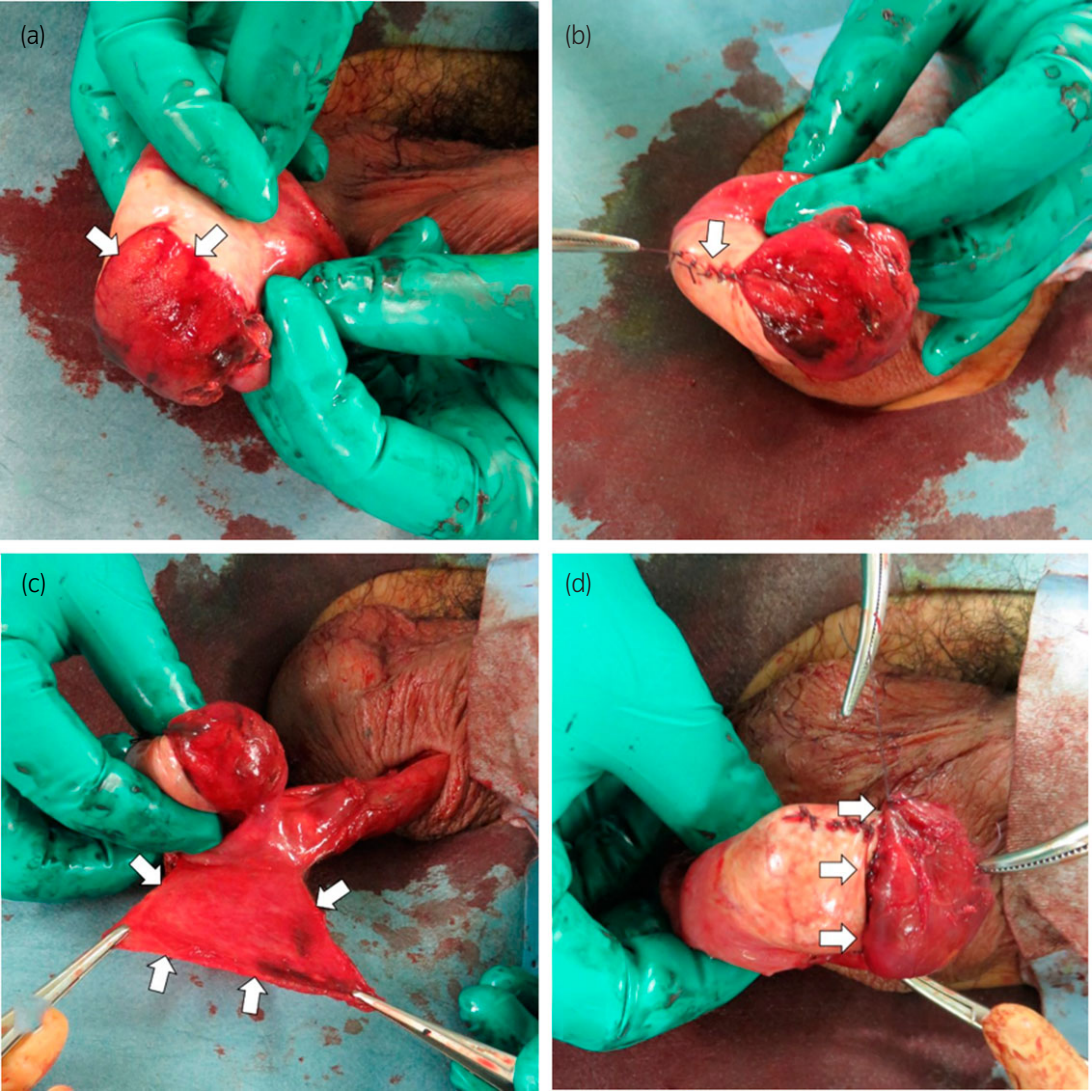
Intraoperative findings. (a) Ruptured tunica albuginea and prolapsed testicular contents. (b) Suture between the layers of the tunica albuginea. (c) Tunica vaginalis flap. (d) Suture of the tunica albuginea and testicular serosal flap.

A dark‐red serum drained after incising the tunica vaginalis. The tunica albuginea was extensively torn and the testicular contents were exposed (Fig. 3a). Primary closure of the tunica albuginea with absorbable sutures was initially attempted but not possible to complete (Fig. 3b) since tight closure could cause secondary damage. A vascular pedicle flap was prepared by shaping the tunica vaginalis (Fig. 3c) to replace the damaged tunica albuginea (Fig. 3d). The dartos fascia and scrotal skin were sutured with absorbable sutures.

### Postoperative course

**Fig. 4:**
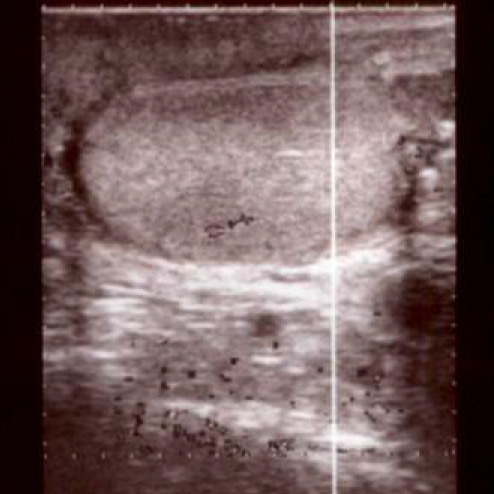
Ultrasonography after 3 months.

The patient was discharged 2 days postoperatively. Ultrasonography identified no abnormalities in the size and blood flow of the ruptured testis at 2 weeks and again at 3 months’ follow‐up (Fig. 4). The LH, FSH, and testosterone levels were 6.7 mIU/mL, 13.8 mIU/mL, and 1065.9 ng/dL, respectively, at 3 months postoperatively. No abnormalities were observed either morphologically or functionally. Thus, the tunica vaginalis flap was considered to successfully preserve the injured testis.

## Discussion

We herein describe a case of extensive rupture of the tunica albuginea and prolapse of the testicular contents with hematoma. There was no necrosis, but the placement of the herniating testicular tissue in the tunica albuginea was considered to be difficult, even if the hematoma was removed. Sacrificing viable seminiferous tubules could have enabled primary closure. However, this method seemed to be inappropriate for this young man in terms of fertility preservation, because forcible primary closure could cause interruption of blood flow and necrosis of the testes, known as testicular compartment syndrome.^10^ Thus, we opted to use a tunica vaginalis flap.

The tunica vaginalis has been used as a substitute tissue in urologic reconstructive surgery.^11^ Its use for repairing a torn tunica albuginea was first reported in 1992 by Kapoor *et al*.^12^ In addition, Ferguson *et al*. reported seven successful cases of testicular repair using the tunica vaginalis after gunshot injuries to the testes.^13^ These reports used free graft patches. Molokwu *et al*. introduced a vascularized flap as an easier and quicker technique,^14^ which was later used by other groups.^15^, ^16^ Damle *et al*. reported that postoperative ultrasound scanning showed no difference in the size, and revealed good blood flow after tunica vaginalis flap repair for testicular injury.^16^

An artificial graft (Gore‐Tex^®^ Patch; W. L. Gore & Associates, Newark, DE, USA) has been trialed previously as an alternative method to cover the defect in the tunica albuginea.^13^ Unfortunately, this graft results in high infection rates, and the authors have stated that this technique should be avoided.

Thus, when primary closure is difficult, substitutes to cover the defect of the tunica albuginea comprise a free graft or tunica vaginalis flap. The success of the free graft depends on the blood flow of the graft bed, and recanalization is essential. In contrast to the free graft, the tunica vaginalis flap is a pedicled substitute and has its own blood flow. To our knowledge, no studies have investigated which is more optimal reconstruction. We prefer flaps to grafts of preserving the blood flow to the substitutes. The influence of the tunica vaginalis flap on the blood‐testis barrier is a point of concern. To our knowledge, no studies have addressed this issue. Further research is required.

## Conclusion

When primary closure of large tunica albuginea defects is difficult, urologists should consider a vascularized tunica vaginalis flap as a potential option. If tension‐free suturing is difficult due to a short pedicle, a free graft can be utilized as an alternative.

## Conflict of interest

The authors declare no conflict of interest.
